# Relationship between Whole-Blood Magnesium and Cognitive Performance among Chinese Adults

**DOI:** 10.3390/nu15122706

**Published:** 2023-06-10

**Authors:** Zijian Lu, Ruikun He, Ying Zhang, Benchao Li, Fengping Li, Yu Fu, Shuang Rong

**Affiliations:** 1 Academy of Nutrition and Health, Hubei Province Key Laboratory of Occupational Hazard Identification and Control, School of Public Health, Wuhan University of Science and Technology, Wuhan 430065, China; 2 BYHEALTH Institute of Nutrition & Health, No.3 Kehui 3rd Street, No.99 Kexue Avenue Central, Huangpu District, Guangzhou 510663, China

**Keywords:** mild cognitive impairment, magnesium, middle-aged and older adults, dry blood spot, cross-sectional study

## Abstract

Objective: To explore the association between magnesium levels and the odds of mild cognitive impairment (MCI). Method: In this cross-sectional study of 1006 participants (≥55 years) from China, whole-blood magnesium concentration was measured using inductively coupled plasma mass spectrometry. MCI was diagnosed according to Petersen criteria using self-reported cognitive decline and a neuropsychological test battery, including the trail-making test-part B (TMT-B), auditory verbal learning test (AVLT), digit symbol substitution test (DSST), and verbal fluency test (VFT), which measured the assessment of executive, memory, attention, and language functioning, respectively. A logistic regression was used to assess the relationship between magnesium levels and MCI, and linear regression analyses were performed for the association between magnesium and cognitive function score. Results: The MCI group had a significantly lower concentration of magnesium compared to the Non-MCI group (34.7 ± 9.8 vs. 36.7 ± 9.7, *p* = 0.017). After adjusting for covariates, a negative association was observed between magnesium levels and MCI. Compared with the lowest quartile (median: 25.4 mg/L), the odds ratio for MCI was 0.53 (95%CI 0.32–0.90) for the highest quartile (median: 48.4 mg/L), and there was an inverse dose–response relationship (*p* for trend = 0.009). In addition, higher levels of magnesium were positively correlated with VFT scores (β = 0.37, 95%CI = 0.11–0.62) and DSST scores (β = 0.50, 95%CI = 0.01~0.98) and negatively correlated with TMT scores (β = −1.73, 95%CI = −3.40–−0.07) in the middle-aged and older adults. Conclusions: Whole-blood magnesium was inversely associated with the occurrence of MCI and positively associated with performance in neuropsychological tests assessing attention, executive, and language ability in middle-aged and older adults.

## 1. Introduction

Cognitive impairment is a serious public health problem worldwide, mainly manifested by a decline in memory, language, and other cognitive functions. According to the World Alzheimer’s Disease Report 2021, more than 55 million people worldwide suffer from cognitive disorders and most of the patients were over 55 years old [1]. Considering the lack of effective treatment for cognitive disorders, it is urgent to distinguish potential risk factors to prevent the occurrence of cognitive disorders. Mild cognitive impairment (MCI) is clinically used to describe a cognitive disorder that is not impairing enough to qualify as dementia, providing a framework for identifying risk factors for cognitive disorders.

Magnesium is a vital micronutrient in the human body, which has a potentially beneficial effect in preventing multiple morbidities [2,3]. In recent years, a growing number of studies have shown that magnesium is positively related to the prevention of cognitive disorders. Experimental studies on cognitive function using animal models have found that higher brain Mg^2+^ levels could improve cognitive function by enhancing short-term and long-term synaptic facilitation [4]. However, human studies on magnesium and cognition are limited and inconsistent. A study of 2508 adults aged 60 and older, which was based on the National Health and Nutrition Examination Survey (NHANES) conducted between 2011 and 2014, showed that higher dietary intake of total magnesium was independently associated with 0.15 higher global cognitive z-score [5]. Nevertheless, a randomized controlled trial including 209 patients who had aneurysmal subarachnoid hemorrhage (aSAH), which administered 64 mmol/day magnesium sulfate daily or placebo during hospitalization intervention, showed that treatment with magnesium has no effect on cognitive outcome after aSAH [6]. One cross-sectional study including 1000 Qatari participants aged ≥20 years old showed that lower serum magnesium concentrations were associated with poorer cognitive function [7]. In the Rotterdam study, both low and high serum magnesium levels were associated with an increased risk of all-cause dementia [8]. Furthermore, the Atherosclerosis Risk in Communities (ARIC) study showed that low midlife serum magnesium was associated with an increased risk of incident dementia but did not affect the rate of cognitive decline [9]. A recent cohort study showed that people with higher levels of magnesium were less likely to develop cognitive disorders compared to people with hypomagnesemia [10].

China has the largest population of cognitive disorders in the world [11]. The primary dietary intake of magnesium includes green leafy vegetables, whole grains, nuts, and legumes. The average dietary intake of magnesium among Chinese individuals was higher than that of Europeans and Americans due to different dietary patterns [12]. Currently, there are no studies on the relationship between magnesium and MCI or cognitive function among Chinese middle-aged and older adults, which needs to be explored. Previous studies have not considered the relationship between cognitive function and magnesium from all cognitive domains. Therefore, our study aimed to investigate the association between whole-blood magnesium concentration and the prevalence of MCI and cognitive function from all cognitive domains in Chinese adults.

## 2. Method

### 2.1. Study Population

**Figure 1:**
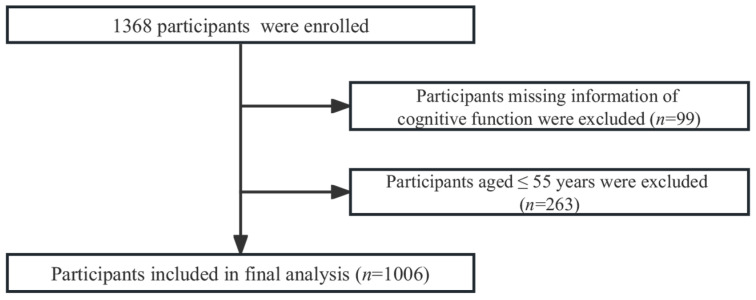
Flowchart showing the inclusion of the study participants.

This study was a cross-sectional study. Participants in this study were recruited through the Health Commission of Hubei Province’s Scientific Research Project and Nutrient Levels of Cognitive Impairment Study. One thousand three hundred and sixty-eight middle-aged and older adults were enrolled from March 2021 to December 2021 in Xiangyang, Hubei Province, China. Participant recruitment was conducted in the community. All participants meeting the following eligibility criteria were included: (i) Chinese; (ii) aged 55 or above; (iii) completed the detection of magnesium concentration using dry blood spot; (iv) completed cognitive function measurements; and (v) no participation in any clinical trial in the three months prior to the survey. Participants who (i) missed any item or component of cognitive tests; (ii) were in the acute phase of severe diseases (e.g., cardiovascular and cerebrovascular diseases, cancer, and psychiatric diseases); and (iii) had a diagnosis of dementia or a Clinical Dementia Rating (CDR) score of ≥1 were excluded. Finally, our analysis included 1006 participants after excluding 362 people with missing information on cognitive function (*n* = 99) and those aged <55 (*n* = 263) (Figure 1).

This study was approved by the Ethics Committee at Wuhan University of Science and Technology (reference number: 201925) and obtained informed consent from all participants. The authors declare that this study complied with the ethics code of the Declaration of Helsinki.

### 2.2. Cognitive Function

A cognitive function survey was conducted face-to-face by professional investigators. The neuropsychological test battery (NTB) was used to diagnose MCI, which included a trail-making test B (TMT-B), auditory verbal learning test (AVLT), digit symbol substitution test (DSST), and verbal fluency test (VFT). TMT asked subjects to connect digits in different colors sequentially and calculate the time spent. The AVLT mainly tests the subjects’ ability to learn words and calculates the correct number of words they recall each time. It required the subjects to first learn 12 words such as “trousers” 3 times, then perform a short-delayed recall at an interval of 5 min, and then perform a long-delayed recall, cue recall, and recognition at an interval of 20 min. The DSST was based on a digital symbol encoding table, where each digit from 1 to 9 corresponds to a symbol. The subjects were asked to match as many symbols to numbers as possible on a coding table for 90 s, with higher scores representing a better cognitive function. The VFT asked subjects to give as many examples as possible in a limited time (usually 1 min) for a certain category, such as “animals”. The NTB test was able to reflect the situation of four primary cognitive domains including executive, memory, attention, and language function. The neuropsychological assessments were standardized for quantifying cognitive function, and z-scores were calculated for each scale. The total score for the NTB was calculated using the formula: NTB = (z_1_AVLT + z_2_DSST + z_3_VFT − z_4_TMT-B)/4. A higher NTB score means better cognitive function. According to Petersen criteria [13], we defined the participant as having MCI for participants with self-reported memory decline in the last year and concomitant declined score (i.e., the score < mean − 1.5 × SD) on any cognitive test (TMT-B, AVLT, DSST, or VFT).

### 2.3. Whole-Blood Magnesium Concentration

Blood samples were collected, stored, and transported in strict accordance with the requirements. All the anticoagulant tubes needed for sampling were randomly sampled in advance and pre-tested for metal contamination. Only when there was no pollution compared with the blank standard were the tubes used for blood sampling. Subjects were asked to fast for 8 h before sampling, and the samples were collected by medical professionals the next morning. We used a single-use disposable lancet to prick the finger and collected four dry blood spots (DBSs) on a single filter paper. Each paper was air-dried overnight at room temperature and then stored independently in a Ziplock bag with a desiccant at −20 °C for one month until detection. DBS samples are stored in the Institute of Nutrition and Health, Wuhan University of Science and Technology. DBS cards were thawed at room temperature before detection. The detection was performed with an ICP-MS (cat. No. 7800ICP, Agilent Technologies, Inc., Santa Clara, CA, USA) by using the method of Lin Y et al. [14]. This method can detect multiple elements in the blood in a short time. DBS samples were treated with DBS-MS 500 (CAMAG, Muttenz, Switzerland), and all samples were analyzed using the Sciex ExionLC system coupled with Sciex Triple Quad 6500 mass spectrometer (Sciex, Framingham, MA, USA). All samples were processed in a specialized cleaning laboratory. DBS samples were processed under laminar flow conditions, except with a semi-automatic hole punch.

### 2.4. Covariates

The demographical information and lifestyle factors were obtained using standardized questionnaires. Demographical information included age (continuous variable), gender (female or male), education level (less than middle school, middle and high school, college or higher), annual per capita income (<CNY 20, CNY 20–40, or ≥CNY 40 thousand per year), marital status (married, single/widowed/divorced), and employment status (retired or paid employment). Lifestyle factors included smoking status (never, past, or current smoker) and alcohol consumption (never, past, or current drinker). The Physical Activity Scale for the Elderly (PASE) was used to determine if a patient’s physical activity in the past week met the recommendations of the 2020 WHO Guidelines on Physical Activity and Sedentary Behavior (below guideline, met guideline) [15]. Body mass index (BMI) was calculated as weight divided by the square of his height (kg/m^2^, <18.5, 18.5–23.9, 24.0–27.9, or ≥28.0). Hypertension was ascertained using self-reporting and/or blood pressure measurement (systolic blood pressure ≥ 140 mmHg or diastolic blood pressure ≥ 90 mmHg). Diabetes was defined as participants with fasting blood glucose (FBG) ≥ 7.0 mmol/L or random blood glucose (RBG) ≥ 11.1 mmol/L and/or self-reporting. These diseases were considered possible confounders.

### 2.5. Statistical Analysis

All data were input and sorted in Epidata. A Kolmogorov–Smirnov test was used to test the normality of continuous variables, and continuous variables conforming to the normal distribution are shown as mean ± standard deviation (SD), and categorical variables are presented as the numbers (percentages). Continuous variables that did not conform to the normal distribution are shown as the median (quartile). A *t*-test was used for continuous variables conforming to the normal distribution, while the Mann–Whitney U test was used for non-conforming variables. The distribution of whole-blood magnesium was not normal; therefore, the values were converted into normal data using the logarithmic transformation and z-transformation before analysis. The whole-blood magnesium concentrations were stratified into quartiles. In order to compare characteristics between quartile samples, a chi-squared test was used for categorical variables and ANOVA was used for continuous variables. The β coefficient for quartiles related to cognitive function score (TMT-B, AVLT, DSST, VFT, and NTB) were calculated using a multiple linear regression model. In addition, we performed subgroup analyses to estimate the agreement when stratified by age (<60 vs. ≥60 years).

The association between whole-blood magnesium and MCI was evaluated using odds ratios (Ors) with 95% confidence intervals (95% Cis), which were calculated using logistic regression, with the lowest quartile being used as the reference category. Three multivariable models were used to perform multivariable analysis. Model 1 was adjusted for gender and age; model 2 was further adjusted for smoking status, alcohol intake, family income, education level, marital status, and physical activity; and model 3 was further adjusted for BMI, hypertension, and diabetes. All statistical analyses were performed using SAS version 9.4 (SAS Institute Inc., Cary, NC, USA), and a two-sided *p*-value < 0.05 was considered statistically significant.

## 3. Results

### 3.1. Demographic Characterization

The characteristics of the participants classified according to MCI status are shown in Table 1. A total of 1006 participants (151 men and 855 women) aged ≥55 years (average 63.7 ± 5.1 years), with a mean magnesium concentration of 36.4 (SD 9.8) mg/L, were included in the analysis. Of all the participants, 16.0% had MCI; 89.4% received more than middle school education; 84.9% were retired; 5.9% were current smokers; 20.0% were current drinkers; 62.8% met the recommended amount of exercise; 54.5% suffered from hypertension; and 7.2% suffered from diabetes.

**Figure 2:**
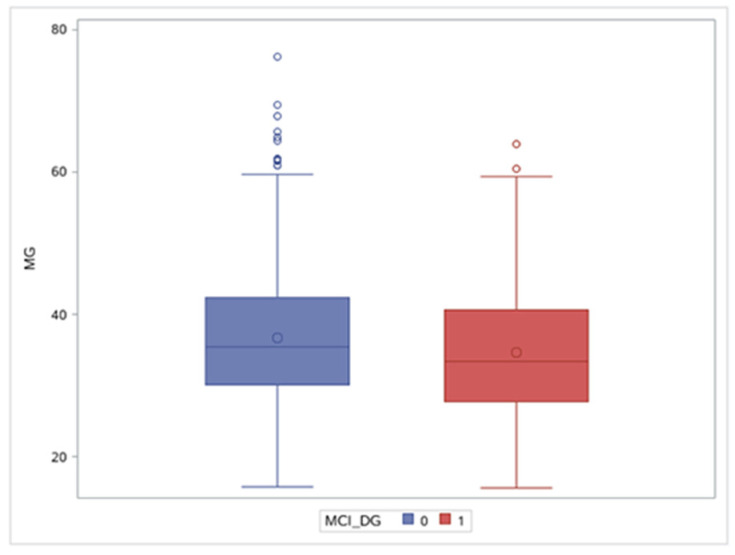
Blood magnesium concentration in the MCI group and the Non-MCI group. MCI: mild cognitive impairment.

Significant differences in magnesium concentration were observed between the MCI group and the Non-MCI group (Figure 2), and the mean magnesium concentration of the MCI group was lower than that of the Non-MCI group (34.7 ± 9.8 mg/L vs. 36.7 ± 9.7 mg/L, respectively, *p* = 0.017). The age of the MCI group was significantly higher than that of the Non-MCI group (66.5 ± 5.2 years vs. 63.1 ± 4.9 years, respectively, *p* < 0.001). The educational level in the MCI group was lower, with 35.4% receiving less than middle school, while the 5.9% of the Non-MCI group receiving this education level. The economic level of the non-MCI group was significantly higher than that of the MCI group: 31.4% of the non-MCI group had an average annual income of more than CNY 4000, while only 16.2% of the MCI group had this income level. As for physical activity, 65.1% of the non-MCI group met physical activity recommendations, while only 50.9% of the MCI group met the recommendations, and the difference was statistically significant. The scores for the MCI group were significantly lower than those for the Non-MCI group on all cognitive screening scales.

### 3.2. Association between Magnesium and Cognitive Function

#### 3.2.1. Magnesium Level and Cognitive Function

**Figure 3:**
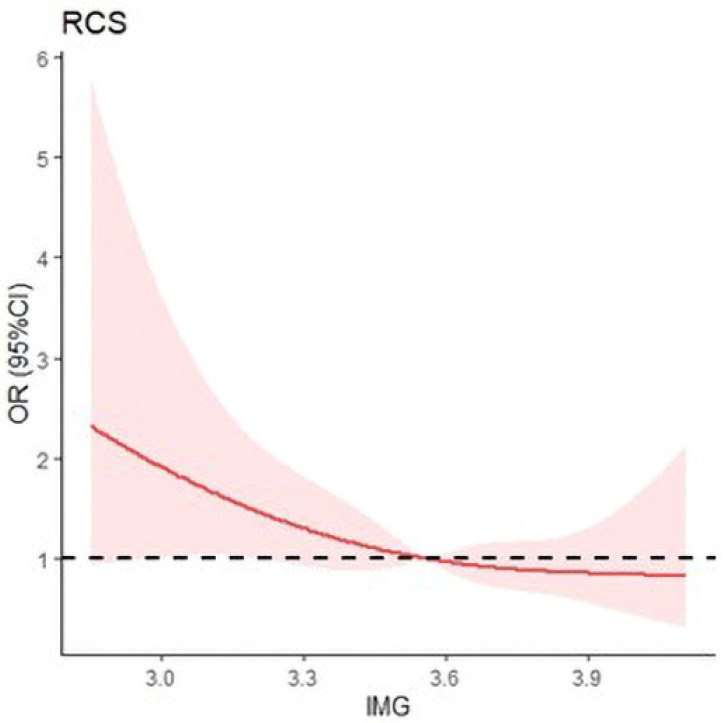
Restricted cubic spline of magnesium and MCI. lMG: The magnesium levels after logarithmic transformation. The model was adjusted for gender, smoking status, physical activity level, alcohol intake, income, education level, physical activity, marital status, BMI, hypertension, and diabetes.

The magnesium concentration was inversely associated with the risk of MCI. The restricted cubic spline for magnesium level and MCI is shown in Figure 3. After adjusting for sociodemographic characteristics and lifestyle factors, as shown in model 3, the Ors (95% Cis) for MCI were 0.44 (95%CI 0.26–0.75), 0.52 (95%CI 0.31–0.87), and 0.53 (95%CI 0.32–0.90) from the second to the highest whole-blood magnesium quartile, respectively (*p* for trend = 0.009), compared with the lowest quartile (Table 2). High magnesium concentration was a protective factor for mild cognitive impairment.

#### 3.2.2. Magnesium Concentrations and Multiple Cognitive Domains

The association between different concentrations of magnesium and each dimension of the cognitive function evaluation is illustrated in Table 3. In the fully adjusted models, higher concentrations of magnesium were positively correlated with VFT scores (β: 0.37, 95%CI: 0.11–0.62), DSST scores (β: 0.50, 95%CI: 0.01–0.98), and z-scores (β: 0.03, 95%CI: 0.02–0.09), and higher concentrations of magnesium were negatively correlated with TMT scores (β: −1.73, 95%CI: −3.40–−0.07) in middle-aged and older adults. The results were analyzed after age stratification, and it was found that high concentrations of magnesium had a protective effect on the general population and people over 60 years old in preventing mild cognitive impairment using the fully adjusted models (Table 4).

## 4. Discussion

In this cross-sectional study, we observed that a high magnesium concentration was inversely associated with the odds of MCI. The effect was not significant below 60 years of age. The magnesium concentration in participants with MCI was significantly lower than that in the Non-MCI group. A high magnesium concentration was positively correlated with the attention, executive, and language ability of middle-aged and older adults.

Our findings are consistent with previous studies on the association between magnesium concentrations and cognition [7,16,17]. Low magnesium concentration is associated with poor cognitive function [7]. The concentration of magnesium in patients with Alzheimer’s disease and mild cognitive impairment was lower than that in normal participants [18,19]. Dietary magnesium intake was also associated with better cognitive functioning [20,21,22]. A prior study found that a higher intake of total magnesium was associated with a significantly higher DSST score [23], which is consistent with the positive correlation between whole-blood magnesium and DSST scores in our study. In addition, in a randomized intervention trial, 25 mg/kg/day Magnesium-L-threonate intervention was shown to have a potential protective effect on cognitive impairment [24]. In another double-blind, placebo-controlled study that included 109 Chinese participants, the results indicated significant benefits of magnesium L-threonate in improving memory and cognition in healthy Chinese adults [25]. Our study revealed the relationship between magnesium and MCI and different domains of cognitive function, suggesting that a higher magnesium concentration is associated with better cognitive function.

Three cohort studies on magnesium and cognition are currently available. Two studies found that low serum magnesium was associated with an increased risk of incident dementia [9], and sufficient magnesium status within the normal range may be beneficial to cognitive health [10]. Only one cohort study from the Netherlands found that low and high serum magnesium levels were both associated with an increased risk of all-cause dementia [8]. It is worth noting that the ARIC-NCS cohort study found that low midlife serum magnesium is associated with an increased risk of incident dementia, but there was no relationship between serum magnesium level and cognitive decline in any cognitive domains including executive function, memory, and language [9]. In this cohort study, the participants were Black and White Americans, and serum magnesium was measured using the Gindler and Heth method [26]. In our study, high magnesium concentrations were positively correlated with attention, executive, and language. The contradictory results may be due to the use of different diagnostic criteria, testing methods, and participants from different regions of the world. In our study, the participants were Chinese middle-aged and older adults, and whole-blood magnesium was used instead of serum magnesium. Other studies have shown that blood releases a significant amount of ionized Mg^2+^ during coagulation; therefore, the concentration of whole-blood magnesium detected using dry blood spots was lower than that in the serum [27]. Although, DBS-LC-MS/MS has been used specifically to store and measure a variety of minerals, including magnesium [28,29], and previous studies have proved the accuracy of DBS-LC-MS/MS [30,31]. Whether the above explanations are the reasons for the difference in study results still needs further investigation.

Several mechanisms can explain the relationship between magnesium and cognition. One hypothesis is the direct regulation of neuronal magnesium on N-methyl D-aspartate (NMDA) receptors. NMDA receptors play important roles in the process of learning and memory formation [32]. Over-excitation of NDMA receptors may weaken synaptic activity and lead to neuronal necrosis [32,33]. Neuronal Mg^2+^ regulates the opening duration and coincidence detection ability of NMDA through pore blocking, thus blocking the excitotoxicity induced by NMDA [34,35]. Another hypothesis is oxidative stress and chronic inflammation. Magnesium deficiency stimulates the secretion of inflammatory mediators such as interleukin-a, tumor necrosis factor-a, and nitric oxide [36,37], leading to higher rates of neurodegeneration and stimulating [38], thereby increasing the risk of dementia. Magnesium has been shown to inhibit the production of excessive amyloid β-protein and prevent this inflammatory cascade [39]. In addition, a magnesium deficiency could determine ECG abnormalities and arrhythmia increasing the risk of atrial fibrillation and stroke and consequently dementia, and hypomagnesemia affects endothelial function at multiple levels. Magnesium deficiency promotes increased production of reactive oxygen species in endothelial cells and vasoconstriction, thus affecting vascular structure and function. Mg^2+^ reduces platelet aggregation and further prevents blood coagulation [40], favoring a pro-atherogenic and pro-thrombotic inflammatory environment that ultimately ends in plaque formation and progression. Vascular disease is the second cause of dementia. These could eventually lead to impairment of cognition [5,41,42,43]. Previous studies have shown that higher vitamin D levels can increase magnesium absorption in the intestine and retention using both animal and human models [42,43]. A recent study found that the positive association between magnesium intake and cognition was primarily among subjects with sufficient serum 25(OH)D level. Further studies are needed to verify this association between magnesium and cognition.

To the best of our knowledge, this investigation is the first to explore the association between magnesium concentrations and the overall global cognition and specific domains of cognitive function in Chinese middle-aged and older adults. We performed the neuropsychological test battery, including executive, memory, attention, and language, which better reflects the effect of magnesium on cognitive function in different fields.

Several limitations of our study should be mentioned. First, we used observational data, and it is difficult to infer causality from these data. Second, considering the number and storage of samples, the dry blood spot was used to detect the concentration of magnesium in the whole blood instead of the serum in this study. In order to compensate for this problem, we used the most accurate detection technology to detect magnesium concentrations; whether this method can truly reflect the level of magnesium in the human body remains to be further studied. Third, the possibility of potential confounders cannot be completely ruled out. Indeed, we only considered the effect of magnesium on cognitive function. Still, the combined effect or interference effect of other micronutrients and magnesium on cognitive function could not be studied. Finally, though we adjusted for many biological and lifestyle factors and undertook the rigorous adjudication of MCI, there may still be some potential confounders that were not considered. For example, we did not rule out the effects of magnesium intake on cognitive function, which potentially inflated the effect of whole-blood magnesium on MCI.

## 5. Conclusions

This study demonstrated that whole-blood magnesium was inversely associated with the occurrence of MCI, and the effect was significant for those over the age of 60. Whole-blood magnesium was positively associated with performance in neuropsychological tests assessing attention, executive, and language ability in middle-aged and older adults.

## Figures and Tables

**Table 1:** Characteristics of participants by MCI status.

	Total	Non-MCI	MCI	*p*-Value ^a^
N (*n*, %)	1006	845 (84.0)	161 (16.0)	
Age, years, mean (SD)	63.7 (5.1)	63.1 (4.9)	66.5 (5.2)	<0.001
Female (%)	855 (85.0)	718 (85.0)	137 (85.1)	0.968
Living alone, *n* (%)	71 (7.1)	57 (6.8)	14 (8.7)	0.747
Marital status, *n* (%)				
Married	870 (86.5)	734 (86.9)	136 (84.5)	0.416
Single/widowed/divorced	136 (13.5)	111 (13.1)	25 (15.5)	
Education level, *n* (%)				
Less than middle school	107 (10.6)	50 (5.9)	57 (35.4)	<0.001
Middle and high school	762 (75.8)	665 (78.7)	97 (60.3)	
College or higher	137 (13.6)	130 (15.4)	7 (4.4)	
Annual per capital income, *n* (%)				
<CNY 20,000	224 (22.3)	177 (21.0)	47 (29.2)	<0.001
CNY 20,000–40,000	491 (48.8)	403 (47.7)	88 (54.7)	
>CNY 40,000	291 (28.9)	265 (31.4)	26 (16.2)	
BMI, *n* (%)				
<18.5	13 (1.3)	9 (1.1)	4 (2.5)	0.056
18.5–23.9	454 (45.1)	392 (46.4)	62 (38.5)	
24.0–27.9	424 (42.2)	356 (42.1)	68 (42.2)	
≥28.0	103 (10.2)	78 (9.2)	25 (15.5)	
Smoking (current), *n* (%)	59 (5.9)	51 (6.0)	8 (5.0)	0.859
Alcohol intake (current), *n* (%)	201 (20.0)	166 (19.6)	35 (21.7)	0.420
Meeting physical activity recommendation, *n* (%)	632 (62.8)	550 (65.1)	82 (50.9)	<0.001
Retired, *n* (%)	854 (84.9)	729 (86.3)	125 (77.6)	0.005
Hypertension, *n* (%)	548 (54.5)	447 (52.9)	101 (62.7)	0.022
Diabetes, *n* (%)	72 (7.2)	61 (7.2)	11 (6.8)	0.862
AVLT, score, mean (SD)	28.8 (9.4)	28.1 (9.6)	18.9 (9.7)	<0.001
VFT, score, mean (SD)	16.6 (4.3)	17.2 (4.1)	13.0 (3.8)	<0.001
DSST, score, mean (SD)	30.6 (8.4)	30.7 (8.4)	18.9 (7.9)	<0.001
TMT-B, seconds, mean (SD)	56.3 (29.1)	49.6 (19.4)	91.6 (42.84)	<0.001
Mg^2+^ (mg/L), mean (SD)	36.4 (9.8)	36.7 (9.7)	34.7 (9.8)	0.017

Data are expressed as the mean (SD) or *n* (%). Abbreviations: SD, standard deviation; MCI, mild cognitive impairment; AVLT, auditory verbal learning test; VFT, verbal fluency test; DSST, digit symbol substitution test; TMT-B, trail-making test B; BMI, body mass index; ^a^ *p*-value for a chi-square test or one-way analysis of variance.

**Table 2:** Association between the level of magnesium and prevalence of MCI.

	Quartile 1	Quartile 2	Quartile 3	Quartile 4	*p* Trend
**Median (IQR) (mg/L)**	25.4 (14.0, 29.6)	32.3 (29.6, 35.1)	38.5 (35.1, 42.3)	48.4 (42.4, 76.2)	
**Model 1**	1 (ref)	0.49 (0.30, 0.81)	0.54 (0.33, 0.87)	0.53 (0.33, 0.86)	0.009
**Model 2**	1 (ref)	0.44 (0.26, 0.74)	0.51 (0.30, 0.85)	0.55 (0.33, 0.92)	0.008
**Model 3**	1 (ref)	0.44 (0.26, 0.75)	0.52 (0.31, 0.87)	0.53 (0.32, 0.90)	0.009

Model 1: Adjusted for age and gender. Model 2: Additionally adjusted for smoking status, physical activity level, alcohol intake, income, education level, physical activity, and marital status based on model 1. Model 3: Additionally adjusted for BMI, hypertension, and diabetes based on model 2.

**Table 3:** Association between the adjusted mean of domain-specific cognitive scores and magnesium concentration.

	AVLT, Score	VFT, Score	DSST, Score	TMT-B, Score	Z-Score, Score
**Model 1**	0.31 (−0.25, 0.98)	0.40 (0.14, 0.66)	0.58 (0.05, 1.12)	−1.90 (−3.59, −0.20)	0.06 (0.02, 0.10)
**Model 2**	0.31 (−0.30, 0.91)	0.37 (0.11, 0.62)	0.49 (0.00, 0.98)	−1.75 (−3.41, −0.09)	0.05 (0.02, 0.09)
**Model 3**	0.31 (−0.29, 0.91)	0.37 (0.11, 0.62)	0.50 (0.01, 0.98)	−1.73 (−3.40, −0.07)	0.03 (0.02, 0.09)

Model 1: Adjusted for age and gender. Model 2: Additionally adjusted for smoking status, physical activity level, alcohol intake, income, education level, physical activity, and marital status based on model 1. Model 3: Adjusted for model 2+ BMI, hypertension, and diabetes.

**Table 4:** Subgroup analysis stratified by an age of 60 years.

	OR (95%CI)		
	Total	<60 Years (*n* = 283)	≥60 Years (*n* = 723)
Mg (mg/L)	0.79 (0.65, 0.95)	0.74 (0.43, 1.28)	0.82 (0.68, 0.98)

Model: Adjusted for gender, smoking status, physical activity level, alcohol intake, income, education level, physical activity, marital status, BMI, hypertension, and diabetes.

## Data Availability

The datasets used to support the findings of this study are available from the corresponding author upon reasonable request.
